# The Impact of a Single Educational Lecture on the Vaccine Confidence among Pregnant Women and Young Mothers

**DOI:** 10.3390/vaccines9030290

**Published:** 2021-03-20

**Authors:** Katarzyna Tkaczyszyn, Ernest Kuchar, Ewa Augustynowicz, Leszek Szenborn

**Affiliations:** 1 Department of Pediatric Infectious Diseases, Wroclaw Medical University, 50-368 Wroclaw, Poland; katarzyna.tkaczyszyn@student.umed.wroc.pl (K.T.); leszek.szenborn@umed.wroc.pl (L.S.); 2 Department of Pediatric Infectious Diseases, University Hospital, 50-556 Wroclaw, Poland; 3 Department of Pediatrics with Clinical Assessment Unit, Warsaw Medical University, 02-091 Warsaw, Poland; 4 Department of Epidemiology of Infectious Diseases and Surveillance, National Institute of Public Health—National Institute of Hygiene, 00-791 Warsaw, Poland; eaugustynowicz@pzh.gov.pl

**Keywords:** education, vaccine, vaccine confidence, visual analogue scale

## Abstract

Background: We investigated the impact of a single unstructured educational lecture about vaccinations on the vaccine confidence in volunteer participants. Methods: We conducted a survey-based study during a series of open meetings related to pregnancy and parenting. Before and after the pediatrician’s lecture related to vaccinations, listeners completed the visual analogue scales (VAS, 0–15 cm), evaluating (1) self-declared knowledge on vaccinations and (2) how they perceive the safety and efficacy of this preventive method. Results: In total, 484 women aged 30 ± 4 years participated in the lecture (pregnant = 68%; ≥1 children = 56%). Participants declared to have more comprehensive knowledge on preventive vaccinations and perceived vaccines to be safer and more useful (the role for the immunity) after vs. before the lecture (median VAS: 10.4 vs. 7.2, 10.8 vs. 8.7, and 11.0 vs. 10.4 cm, all *p* < 0.001). Importantly, the prevalence of vaccine-related adverse events was also assessed as being higher after the lecture (median VAS: 9.9 vs. 8.0 cm, *p* < 0.001). The increase in self-declared knowledge on vaccinations and perceived need for vaccinations (delta VAS—VAS after minus before the lecture, expressed as % of baseline) was lower among participants who rated the lecture less vs. more useful. Importantly, both participants who liked vs. did not like the lecture comparably rated vaccines safer after vs. before the lecture (delta VAS (median, interquartile range): 16% (0–39%) vs. 18% (2–42%), *p* = 0.39). Conclusions: An educational lecture on vaccinations positively impacts vaccine confidence in young adult women. Irrespective of the subjective rating of the lecture, all listeners perceived vaccinations to be safer after vs. before the speech.

## 1. Introduction

Despite evidence-based diverse medical and socioeconomic benefits of contemporary vaccination schemes (for both particular persons and the whole populations) [1], there are substantially growing public concerns worldwide regarding the efficacy and safety of active immunization procedures, in particular due to disinformation spread by anti-vaccination movements [2,3,4]. The number of “vaccination-hesitant individuals” has grown significantly worldwide and overall vaccine confidence has decreased [5,6,7,8,9]. Many different countermeasures have been implemented to improve immunization rates, ranging from educational campaigns providing reliable medical information to the penalization of mandatory vaccination refusals [10]. Furthermore, concepts of institutional restrictions for unvaccinated children have emerged, such as limitations in terms of access to public education and institutional care (e.g., inability to apply to a public nursery). Recently, the problem of vaccine hesitancy has become discussed in public in the context of the coronavirus disease 2019 (COVID-19) pandemic. There are concerns regarding whether the skepticism of many people towards vaccination against COVID-19 will hinder the achievement of herd immunity, and therefore whether suboptimal vaccination rates will constitute a significant obstacle to better epidemic control worldwide. Cost-effective methods to improve vaccine confidence are constantly being sought to improve vaccination rates [11].

In the current study, we aimed to assess the impact of a single unstructured educational lecture on vaccinations (performed by a pediatrician) on comprehensive opinions regarding this immunization method in volunteer lecture listeners. We hypothesized that the participation in such lectures may positively influence vaccine confidence in the listeners.

## 2. Materials and Methods

We conducted a survey-based study during a series of open meetings (Academy: Mother Asks), arranged and conducted for educational and advertising purposes by a commercial company Academy Mother Asks from Poznan, Poland (meeting organizer was not an academic institution) in 11 large Polish cities in 2014. The aim of each educational meeting was to familiarize the volunteers with different topics related to pregnancy, childbirth, and parenting, which were covered by professional educational lectures. Meetings were free of charge for volunteer listeners but included advertising and promotion of commercial services and goods. During each meeting, one educational lecture was related to vaccinations and was performed by a specialist pediatrician with long-term experience in vaccinology. The pediatrician–lecturer received the honorarium for the lecture paid by the organizers. Particular vaccine formulations were not advertised during the meeting.

For the current study, we developed an original Polish-language survey to evaluate the knowledge on vaccinations and the attitudes towards this preventive method before and after the educational lecture. The meeting organizer approved the use of the survey mentioned above for non-commercial scientific purposes and was neither involved in preparing the survey, nor influenced its final content. The survey was completely anonymously (written informed consent was, therefore, not obtained from study participants) and comprised two parts: the first part included questions about gender, age, place of living, and children; in the second part of the questionnaire, study participants were asked to use 0–15 cm visual analogue scales (VAS) to answer six questions regarding the following topics:

1 Participant knowledge on vaccinations (lack of knowledge versus comprehensive knowledge);
2 Participant opinion on the need for vaccinations (no need vs. indisputable, obvious need);
3 Safety of vaccinations (low vs. high);
4 Incidence of adverse events after vaccinations (rare vs. frequent);
5 The role of vaccinations for the immunity of a child (non-significant vs. significant);
6 The role of the disease per se for the immunity of a child (non-significant vs. significant).

English translation of the survey is presented in File S1 to this paper. After answering the questions for the first time, the educational lecture was started and lasted about 60 min. Although the lecture was not structured in general (no strict guidelines were applied), pediatrician–lecturers were asked to review the epidemiology of vaccine-preventable diseases and the critical role of vaccinations for public health. They explained technically what a vaccine is, which vaccines are available, and which adverse reactions are most frequent. All speakers were trained by the meritorical coordinator—an expert in the field of pediatric infectious diseases and vaccinations in children. Additionally, for the purposes of the lecture, pediatricians participating in the initiative were given a set of exemplary slides that could be used in their educational speech. The lecture was presented in plain language (participants were not healthcare professionals—HCPs: physicians, nurses, etc.), and there were opportunities to ask questions during and after the lecture. Beyond general topics and the suggestions mentioned above, the lecture’s content was not rigidly imposed and was at the discretion of the particular lecturer.

After the lecture, participants were asked to complete the same questionnaire as before (only VAS, without the sociodemographic questions answered at the beginning of the lecture). The additional VAS referred to the subjective rating of the lecture (useless vs. very useful—“how do you assess the usefulness of the issues presented in the lecture”).

This study was approved by the Bioethics Committee of Wroclaw Medical University, Wroclaw, Poland.

## 3. Statistical Analyses

Based on the character of particular questions, the VAS scale results (0–15 cm) showed either normal (e.g., knowledge on vaccinations) or obviously skewed distributions (e.g., the significance of vaccinations), and are presented as medians with lower and upper quartiles (an interquartile range, IQR). Hence, for further statistical analyses, we used non-parametric tests. The inter-group differences were tested using the Mann–Whitney U-test for unpaired samples. The VAS results after vs. before the lecture were tested using the signs test. To compare changes in opinion regarding particular aspects of vaccinations in subjects who subjectively rated the lecture better vs. worse, we calculated coefficients delta VAS for particular questions from the survey (delta VAS = VAS after minus VAS before the lecture, expressed as % of VAS before the lecture (baseline)). Categorized variables are expressed as numbers and proportions (%) and the inter-group differences were tested using the Chi-square test.

Statistical analyses were performed using STATISTICA 13.3 data analysis software (TIBCO Software, Inc., Palo Alto, CA, USA).

## 4. Results

### 4.1. Baseline Characteristics and Sociodemographic Data of Female Lecture Participants

There were only a few male participants in the lecture, therefore we did not include them in further statistical analyses, as the potential analysis of gender-related differences would be unreliable due to significant disproportion between women and men. Finally, 484 women participated in the lecture from January to May 2014, who were included in the statistical analyses presented below. They were aged 30 ± 4 years (median: 29) and the majority had a high education level (88%). As many as 81% of female participants declared active employment and over 90% were city residents. The majority of women (68%) were pregnant at the time of the lecture, while 56% had one or more children. Only 9 (2%) women analyzed in this study were neither pregnant nor had children. Baseline vaccine confidence (self-declared knowledge of vaccinations and opinions on the safety and efficacy of this preventive method before the lecture (baseline VAS scales)) according to socioeconomic data are presented in Table 1.

### 4.2. The Impact of the Lecture on the Attitudes towards Vaccinations (VAS Scales)

**Figure 1:**
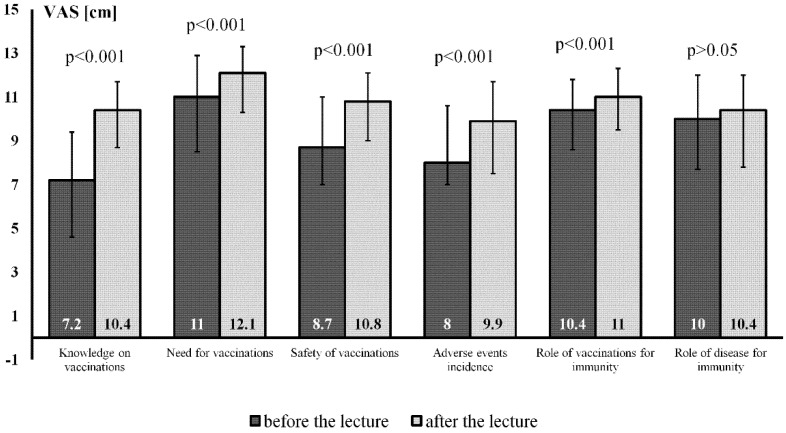
The attitudes towardsvaccinations (visual analogue scales 0–15 cm) before and after the lecture in female lecture participants (medians (boxes) and interquartile ranges (whiskers)). For details, see the Methods section and File S1.

Lecture listeners declared more comprehensive knowledge on and the need for vaccinations after vs. before the lecture (Figure 1). Moreover, after the lecture, the female participants perceived this method to be safer and more useful (regarding the role of immunity), however they also declared more adverse events after vs. before the lecture (Figure 1).

### 4.3. Attitude towards Vaccinations according to the Rating of the Lecture

The median evaluation (rating) of the lecture (the question of whether the lecture was useful and if the listener will use the knowledge learned; VAS scale) was 12.2 cm. The 2 groups (lecture evaluation VAS≤ vs. >median) did not differ regarding age (30 ± 4 vs. 30 ± 4 years, *p* = 0.6). Lecture participants who rated the lecture lower (VAS evaluation ≤ median, i.e., 12.2 cm) had lower baseline knowledge on vaccinations (7.1 (4.5–8.5) vs. 7.5 (4.9–9.7) cm, *p* = 0.03) and perceived this method to be less safe (7.6 (6.2–10.1) vs. 9.8 (7.7–11.6) cm, *p* < 0.001), less useful (regarding the need for vaccinations, 10.2 (7.3–12.3) vs. 12.0 (10.3–14.0) cm, *p* < 0.001), and less efficient (role of vaccinations for immunity; 9.7 (7.5–11.4) vs. 11.0 (9.7–12.3) cm, *p* < 0.001). Importantly, lecture listeners who finally evaluated the lecture less useful declared at baseline (before the lecture) that vaccinations are related to lower incidence of adverse reactions as compared with subjects who liked the lecture and assessed it as more useful (7.7 (6.8–10.0) vs. 8.9 (7.3–11.0) cm, *p* < 0.001).

The changes in self-declared knowledge on and the need for vaccinations (delta VAS—i.e., VAS after the lecture minus before the lecture (cm), expressed as % of baseline VAS (before the lecture)) were significantly lower among participants who perceived the lecture as less vs. more useful (delta VAS: 27% (6–77%) vs. 43% (19–118%), *p* < 0.001; 3% (5–21%) vs. 5% (0–20%), *p* = 0.02; respectively). Importantly, the change in opinion on the safety of vaccinations was comparable in subjects who liked vs. did not like the lecture (both groups perceived vaccinations as comparably safer after the lecture; delta VAS: 16% (0–39%) vs. 18% (2–42%), *p* = 0.39). The difference regarding the change in awareness of adverse reactions to vaccines (frequency of such reactions evaluated using VAS) was of borderline statistical significance (delta VAS: 4% (−5–27%) vs. 9% (−3–36%), *p* = 0.06).

## 5. Discussion

There were two major findings arising from our survey-based study. Firstly, we demonstrated that a single unstructured educational lecture positively impacts vaccine confidence in young adult women (mostly pregnant or with children). Secondly, irrespective of the subjective rating of the lecture, both listeners who rated the lecture higher vs. lower perceived vaccinations as safer after vs. before the lecture.

In 2019, the World Health Organization considered vaccine hesitancy as one of the major threats to global health [12]. Although the vaccination coverage rate in Poland is still as high as approximately 90% (it is worth noting that this value relates to mandatory vaccinations), the number of intentional vaccine refusals has increased significantly—from 5340 in 2012 to 39,785 in 2018 [13]. These findings correspond with both European and global data, demonstrating the renaissance of vaccine-preventable diseases, e.g., from January to April 2019, there were 704 cases of measles reported in the United States (incidence of measles directly correlates with vaccine refusals), which means the highest number of cases reported in 25 years [3,14].

Different factors influencing the vaccine confidence in particular persons and the whole populations or communities have been the subject of research interest in recent years, including societal, economic, and psychological determinants. [3,9,15]. It needs to be emphasized that the comprehensive knowledge of the parents or caregivers on vaccinations constitutes the major determinant of vaccine confidence [15,16,17,18,19,20]. According to the recent State of Vaccine Confidence in the European Union in 2018 [3], the public perceptions of vaccination were mostly positive, and over 70% of Poles declared that they recognized the importance and safety of and had trust in vaccinations. It is worth noting, however, that in Poland there has been the highest decrease in vaccine confidence since the report in 2016 [3]. Decreasing vaccine confidence in Poland may be partially explained by the rapid development of anti-vaccination movements, which deter parents and caregivers from vaccinating children and promote unwillingness towards active immunization [3]. Indeed, in one large study comparing vaccine confidence in 18 European countries, the highest levels of confidence were in Portugal and Cyprus, while the lowest were reported in Poland and Bulgaria. The authors noticed that most parents (83%) received negative information on vaccinations, mainly from the Internet, which made them more hesitant and more likely to refuse vaccinations [21].

Importantly, the accumulated evidence demonstrates that for parents the direct contact with a physician (pediatrician) who reliably informs about vaccinations plays a key role in improving comprehensive knowledge on vaccinations and overall vaccine confidence [9,10,21,22,23,24]. Indeed, the education of physicians further improves vaccine confidence in managed families (children+parents) [21]. It is still unknown, however, which educational and social interventions are most cost-effective in terms of reversing the aforementioned negative trends regarding decreasing social trust towards either vaccinations or healthcare professionals involved in immunization procedures and managing vaccination programs (e.g., general practitioners, pediatricians). Hence, numerous educational campaigns have been conducted worldwide to objectively inform parents about the role of preventive vaccinations, in order to present the possible detrimental consequences of the resurgence of particular vaccine-preventable diseases, and eventually to improve general vaccine confidence [25,26,27,28,29].

Our study contributes to previous observations that education is the key determinant of vaccine confidence. We have demonstrated that a simple, single educational intervention (open lecture) positively affects the perception of the safety of vaccinations and their role for public health in young adult women. Such interventions may further decrease the number of vaccine-hesitant individuals [25,28,30,31], however the long-term effects of such educational campaigns or meetings, as well as the precise cost-effectiveness, require further investigation. It needs to be acknowledged that the positive changes in the perceived safety of vaccinations were similar in groups with lower vs. higher subjective ratings of the lecture. As the perception of vaccine safety constitutes the key element contributing to overall vaccine confidence [21], this observation highlights the uniform educational value of a direct lecture given by an experienced healthcare professional in terms of decreasing public concerns regarding vaccinations. It is, however, unknown whether such uniform educational effects (independent of the rating of the lecture) are also valid for television or Internet campaigns. It also needs to be acknowledged that HCPs are still the most reliable and respected source of information on preventive vaccinations and can impact the attitudes of parents and patients towards active immunization the most. For example, Czajka et al. demonstrated that people who were not thoroughly informed about vaccinations by HCP, but rather obtained information from another source (media, the Internet, acquaintances), were twice as likely to be unconvinced by this method of active immunization compared with those educated by HCP [24]. Indeed, parents’ decisions to vaccinate their children critically depend on receiving reliable knowledge about vaccinations from HCPs [32]. Moreover, when informing about the vaccination procedure, HCPs also have the opportunity to inform parents about potential diverse post-vaccine reactions or side effects, which occur with differing frequency but which are usually mild. They may be associated with temporary discomfort for patients, however with appropriate “mental” preparation (information), they will be considered natural and will not affect the perception of vaccine safety. Parents who receive the above information about possible vaccine-related symptoms and how to deal with them from HCPs are able to accept them, which has a positive effect on their general attitude towards vaccinations. The need to pay special attention to the balance of benefits and risks for the child is also emphasized—adverse post-vaccination reactions vs. disease and its possible complications [18,22,23,24,32].

However, attention is drawn to the fact that often a lack of time, overload with work and duties, and a lack of specific training (how to effectively educate, shape opinions, and influence the change of views, also in regarding psychological aspects) have negative impacts on HCPs, who are not able to adequately answer all of the parents’ questions or address particular doubts and fears, especially in the face of rapidly changing medical recommendations, as well as with diverse new preparations appearing on the market (e.g., sophisticated combined vaccines) [22]. This is especially evident in the context of the current coronavirus disease 2019 (COVID-19) pandemic. Twelve months after the first cases of this completely new infectious disease syndrome were described, new-generation vaccines were approved for use in the European Union based on messenger ribonucleic acid or vector technologies, which both raised hopes that we will be able to control the pandemic, but also fear and doubts regarding whether such preparations are safe in the long term. It needs to be pointed out once again that the success of the COVID-19 vaccination campaigns and overcoming the pandemic will critically depend on HCPs and their understanding of the beliefs and fears of patients, along with the appropriate provision of information and effectively adapted educational activities [33].

## 6. Study Limitations

Certain limitations of this study need to be acknowledged. Firstly, the lecture was not structured *a priori* and there were probably differences in the time periods devoted to specific topics during each presentation. Secondly, we measured the changes in opinion on vaccination directly after the lecture, and no follow-up was available to assess the long-term effectiveness of such educational interventions. Finally, most women participating in the study were pregnant, and their opinions on preventive vaccinations may vary from the general population.

## 7. Conclusions

In a survey-based study, we have demonstrated that a single unstructured educational lecture on vaccinations positively impacts the opinions on this preventive method in young adult women, most of whom were pregnant or had children. Importantly, irrespective of the subjective rating of the lecture, both listeners who rated the lecture higher vs. lower perceived vaccinations as safer after vs. before the lecture. Further studies evaluating individual and social benefits of such educational actions or events (including follow-up) are warranted to precisely evaluate the cost-effectiveness.

## Figures and Tables

**Table 1:** Baseline self-declared knowledge of and attitudes towards vaccinations (visual analogue scales) according to sociodemographic data.

Survey Element	Age ^#^		Educational Level		Active Employment		Children		Pregnancy	
	≤29 Years (*n* = 248)	>29 Years (*n* = 230)	Primary or Secondary (*n* = 56)	Higher (*n* = 422)	No (*n* = 88)	Yes (*n* = 386)	No (*n* = 266)	Yes (*n* = 211)	No (*n* = 150)	Yes (*n* = 326)
VAS question 1 Self-declared knowledge of vaccinations (cm) (more cm = greater knowledge)	7.1 (4.4–9.05)	7.4 * (5.2–9.6)	5.5 (3.35–9.4)	7.2 (5.0–9.4)	7.0 (4.5–8.3)	7.2 (4.7–9.5)	5.65 (3.4–7.5)	8.5 ** (7.1–10.1)	8.35 (7.1–10.1)	6.5 ** (3.9–8.2)
VAS question 2 Need for vaccinations (cm) (more cm = greater need)	11.05 (8.7–13.0)	11.0 (8.3–12.7)	12.0 (9.15–13.15)	11.0 (8.3–12.8)	11.55 (8.6–13.0)	11.0 (8.4–12.9)	11.0 (8.4–12.8)	11.25 (8.7–13.0)	11.6 (8.9–13.1)	11.0 (8.3–12.7)
VAS question 3 Safety of vaccinations (cm) (more cm = more safe)	8.45 (7.0–11.0)	9.1 (6.85–10.9)	8.5 (7.5–10.8)	8.8 (6.8–11.0)	9.5 (7.4–11.1)	8.5 (6.8–11.0)	8.0 (7.0–10.8)	9.1 (6.8–11.2)	9.4 (6.9–11.2)	8.4 (7.0–10.9)
VAS question 4 Incidence of adverse events after vaccinations (cm) (more cm = lower incidence)	8.2 (7.0–10.7)	7.95 (7.1–10.6)	7.7 (6.8–10.2)	8.2 (7.1–10.7)	8.1 (6.9–10.6)	8.0 (7.1–10.7)	7.95 (7.1–10.6)	8.2 (7.0–10.8)	8.6 (7.1–11.0)	7.8 (7.0–10.4)
VAS question 5 The role of vaccinations for immunity (cm) (more cm = greater role)	10.4 (8.4–12.0)	10.4 (8.7–11.8)	10.7 (9.0–12.05)	10.4 (8.55–11.8)	10.9 (9.2–12.2)	10.3 (8.5–11.8)	10.3 (8.2–11.7)	10.5 (9.0–12.0)	10.7 (9.2–12.1)	10.2 ** (8.2–11.6)
VAS question 6 The role of the disease in immunity (cm) (more cm = greater role)	10.0 (7.6–11.7)	10.1 (7.7–12.4)	9.7 (7.5–12.6)	10.1 (7.7–12.0)	10.4 (8.0–11.9)	10.0 (7.6–12.1)	10.0 (7.7–12.2)	10.2 (7.7–12.0)	10.2 (7.7–12.0)	10.0 (7.7–12.0)

Data are presented as medians and interquartile ranges (in parentheses). Note: ^#^ age groups are based on the median age of the studied subjects (29 years); *p*-value legend: * *p* < 0.05, ** *p* < 0.001. For details, please see Methods section and File S1 (English version of the survey, which was originally in Polish).

## Data Availability

Study database is available on request at the Department of Pediatric Infectious Diseases, Wroclaw Medical University.

## Supplementary Materials

The following are available online at https://www.mdpi.com/2076-393X/9/3/290/s1, File S1: Survey.
