# Supplementary material for: The Impact of a Single Educational Lecture on the Vaccine Confidence among Pregnant Women and Young Mothers
Source: Vaccines (Basel). 2021 Mar 20;9(3):290. doi: 10.3390/vaccines9030290 (PMC8003617; doi:10.3390/vaccines9030290)
Supplement: Supplementary file 1 [file vaccines-09-00290-s001.pdf]

File S1: SURVEY

1st part. Please answer the questions BEFORE the lecture about vaccination:

1. Sex: € female € male

2. Age [years]: ……….

3. Education: € primary € secondary € higher

4. Active employment? € yes € no

5. Living place? € city € village/small town

6. Having children? € yes € no

7. How many children do you have?: ……….

8. Currently pregnant? € yes € no

<u>In the following questions, please mark a single line on the vertical line:</u>

9. How do You assess your knowledge on vaccinations?

I know nothing ______________________________ I know everything

10. How do you assess the need for vaccinations to your child?

Unnecessary ______________________________ Absolutely necessary

11. How do you assess the safety of vaccinations?

Very dangerous ______________________________ Very safe

12. In Your opinion, how do you assess the incidence of adverse post-vaccination reactions?

Every time ______________________________ Never

13. In Your opinion, how do you assess the role of vaccinations for the immunity of a child:

Non-significant ______________________________ Significant

14. In Your opinion, how do you assess the role of infectious disease for the immunity of a child:

Non-significant ______________________________ Significant

2nd part. Please answer the questions AFTER the lecture about vaccinations:

<u>In the following questions, please mark a single line on the vertical line:</u>

1. How do you assess your knowledge on vaccinations?

I know nothing	I know everything

2. How do you assess the need for vaccinations to Your child?

Unnecessary	Absolutely necessary

3. How do you assess the safety of vaccinations?

Very dangerous	Very safe

4. In Your opinion, how do you assess the incidence of adverse post-vaccination reactions?

Every time	Never

5. In Your opinion, how do you assess the role of vaccinations for the immunity of a child:

Non-significant	Significant

6. In Your opinion, how do you assess the role of infectious disease for the immunity of a child:

Non-significant	Significant

7. How do you assess the usefulness of the issues presented in the lecture?

Totally useless, I did not like the lecture	Really useful, I liked the lecture
